# Cataract Surgery in Patients with Keratoconus: Pearls and Pitfalls

**DOI:** 10.2174/1874364101711010194

**Published:** 2017-07-31

**Authors:** F. Aiello, QJ Nasser, C. Nucci, R.I. Angunawela, Z. Gatzioufas, V. Maurino

**Affiliations:** 1 Moorfields Eye Hospital, NHS Foundation Trust. London – United Kingdom; 2 Department of Experimental Medicine and Surgery, University of Rome “Tor Vergata” Rome - Italy

**Keywords:** Keratoconus, Cataract surgery, Biometry, Toric IOL, Pearls, Collagen

## Abstract

**Background:**

Keratoconus (KC) is a common ectatic disorder resulting in progressive corneal thinning and irregular astigmatism. It has been observed that patients affected by KC are more likely to develop lens opacities earlier compared to non-keratoconic patients.

**Objective:**

Intraocular lens (IOL) selection and refractive outcome prediction are among a number of factors that can make cataract surgery in keratoconic patients challenging. Accurate biometry is often difficult to obtain due to unreliable K measurements and lack of dedicated biometric formulae. The use of toric IOLs has also been investigated.

**Conclusions:**

Determining the stage of KC, pre-operative patient counselling and the preferred method of refractive correction are all crucial to obtain successful postoperative outcomes and good patient satisfaction. The use of toric IOLs can achieve good results only in selected low-grade keratoconic eyes.

## 1 INTRODUCTION

Keratoconus (KC) is a non-inflammatory corneal dystrophy resulting in progressive corneal thinning and steepening. These corneal changes are responsible for an increase in irregular astigmatism and keratometric myopia resulting in blurred vision [1]. The prevalence of the disease has been estimated to be between 29 and 229 individuals per 100, 000 according to population studies [2-5]. The condition usually begins at puberty, progresses at variable speed over 10-20 years and then stabilizes before the 40’s [1]. Consequently, KC management is mainly determined by the grade of disease and patient age with a treatment aim of both visual improvement and/or disease stabilization [1].

The majority of early KC patients are treated with glasses or with rigid gas permeable (RGP) contact lens (CL) in order to improve the visual acuity. In the late stages of the disease where good visual acuity is limited by poor CL visual performance, corneal scarring or CL intolerance, corneal transplantation becomes the only feasible therapeutic approach, with up to 20% of affected individuals requiring a corneal graft [6, 7].

Prevention of KC progression is important as it may reduce the need for keratoplasty especially in young patients and the morbidity associated with more advanced KC disease [8]. Thus far corneal collagen cross-linking has been shown to be the only treatment option capable of slowing down or halting KC progression [9]. Although nearly all patients will eventually develop visually significant cataract, this tends to occur at a younger age in patients with KC compared with the general population [10]. Cataract surgery in patients with KC is challenging due to the difficulties associated with interpreting keratometry readings, determining the amount of astigmatism, measuring axial length and thus obtaining an accurate IOL power estimation. In addition, cataract surgery in keratoconic eyes can be more technically demanding due to corneal thinning and scarring.

This article aims to analyze the pre- and intra- operative problems that surgeons may encounter when approaching a keratoconic patient requiring cataract surgery.

## 2 PREOPERATIVE ANALYSIS

All currently available biometry formulas require the axial length and keratometry as minimum parameters to evaluate the total eye’s refractive power. In keratoconic eyes the keratometry is the most challenging parameter to measure due to the irregular corneal shape and K values, which vary significantly between those at the cone apex and those from the remaining cornea. Clinical reports have indicated that corneal topography measurements are more variable for steeper corneal curvatures and in the presence of corneal irregularity and scarring [11]. In addition, the reliability of corneal topography and K measurements in KC patients can be adversely affected by fixation difficulties and a displaced visual axis during measurements [12]. Furthermore, it has been well described that each keratometer machine has its specific repeatability that decreases in keratoconic eyes [11, 13, 14].

Hassan Hashemi *et al.* [13] reported the repeatability of keratometry measurements with five different devices (Pentacam, Eyesys, Orbscan, IOLMaster, Javal manual keratometer) in eyes with different grades of KC. The study enrolled 78 eyes of 45 keratoconic patients with no history of eye surgery or contact lens use at least one month before their scans. Three measurements were taken for each device recording the keratometric value on flat and steep axis. Keratometry repeatability was investigated in three different groups according to KC severity: group 1 composed by 27 eyes with maximum K value less then 50.0 diopters (D); group 2, 26 eyes with maximum K value between 50.1-55.0 D; group 3, 25 eyes with maximum K value greater then 55.1 D. Results showed that in patients with K values up to 55.0 D keratometry readings had good repeatability among all the devices with better results in group 1 compared with group 2. The Pentacam device had the highest repeatability while the Orbscan II device the lowest. In Group 3 (K> 55.1D) all 5 devices had low repeatability. Similarly, Yagci R *et al.* [14] evaluated two newer devices for corneal topography repeatability in keratoconic eyes compared with a control group of normal patients. In this study, the Galilei dual Scheimpflug analyzer (Galilei DSA; Ziemer) and Nidek AL Scan (Nidek CO, Aichi, Japan) biometry machines were assessed. The Nidek AL Scan is an optical partial coherence interferometry-based ocular biometry device that analyses the corneal curvature radius (refractive power) over 2.4 and 3.3 mm diameters. The Galilei DSA associates a dual rotating Scheimpflug cameras with a Placido disc for the anterior segment evaluation with more than 122,000 data points measured per scan. Sixty-two healthy eyes and 88 eyes with keratoconus were enrolled in the study. The intra-device repeatability of Ks readings of both devices was excellent in normal and keratoconic groups although in eyes with stage III KC repeatability was worse compared with stage I and II. However, K readings (K flat, K steep, and K medium) taken with the Nidek AL Scan were significantly higher than those taken with the Galilei DSA (P < 0.05). As expected, both devices demonstrated worse repeatability in more-advanced KC stages. These results highlight the difficulty in obtaining reliable values in K measurement that can be safely used in biometry calculation.

Mcmahon *et al.* [11] compared the precision of three Placido corneal topography instruments (Dicon CT 200, EyeSys Model II and Keratron Corneal Analyzer) in keratoconic patients. Sixteen eyes of 9 patients were enrolled and 4 scans were acquired for each eye. The study established that measurement variability (in dioptres) was significantly larger among KC patients compared with normal subjects using similar devices. Furthermore, the repeatability and the accuracy of the scans were reported to be low for all three instruments. The above reports of poor repeatability and accuracy in Ks measurements among different devices elucidates the problem of poor reliability of K values available for the biometric calculation.

Thebpatiphat *et al.* [10] assessed different biometry formulas for IOL calculations in eyes with KC undergoing cataract surgery. Twelve eyes of 9 patients were enrolled in the study. Severity of KC was recorded according to measured K values: <48 dioptres (D); between 48-52 D and >52D corresponding to mild, moderate and severe KC respectively. The authors retrospectively evaluated the difference between intended and achieved refractive outcome applying three different biometry formulas; SRK, SRKII, or SRKT. Overall it was found that the SRK II formula provided better accuracy for IOL power calculation in patients with mild keratoconus. For patients with moderate and advanced KC, IOL calculation was less accurate with no significant difference between calculation formulas found. In standard biometry calculation, many values remain an assumption; in particular it is presumed that Ks measured are the same at the steepest point and at the visual axis and that the posterior corneal surface consistently has a radius of curvature 1.2 mm steeper than the anterior corneal radius [15]. However, these assumptions are not valid for eyes with KC and therefore can lead to inaccurate IOL calculation [16]. The disparity between the anterior and posterior corneal surfaces is not only greater than in normal eyes but also K values and both anterior and posterior corneal surface can be completely irregular in eyes with KC which has an unpredictable impact on all IOL power calculation formulas [17]. Furthermore eyes with KC tend to have longer axial lengths and deeper anterior chambers [18], making the effective estimated lens position less accurate compared with normal eyes [19].

All these variables contribute to significantly less reliable IOL calculations and should be taken into account during the pre-operative patient counselling. The work of Watson *et al.* [19] in a retrospective study evaluated the refractive outcome of keratoconic patients undergoing cataract surgery at Moorfields Eye Hospital, London. The authors reviewed 126 eyes of 91 patients who underwent surgery between 1996 and 2010. Ninety-two eyes (64 patients) met the inclusion criteria for analysis. All eyes were divided into three groups accordingly the grade of disease: K<48D, K between 48-55 D and K>55 were classified as mild (35 eyes), moderate (40 eyes) and severe (17 eyes) respectively. In the mild and moderate group, K values from optical biometry calculation (IOL Master – Carl Zeiss) were used. In the severe group, a standard K value of 43.25D was used for 9 eyes and optical biometry derived Ks for 8 eyes. Postoperative outcome measures included biometry prediction error (BPE) and postoperative refraction correction needs (spectacles or contact lenses). The BPE was defined as the difference between the planned refraction determined by biometry and the spherical equivalent of the final refraction.

In the mild group, the mean BPE was 0.0 D (range: 5.2 D to −3.0 D) with 60% of eyes achieving a postoperative spherical equivalent error within ±1 D of the intended refractive outcome. Four patients changed their preferred refraction error correction from glasses to contact lenses after surgery, 8 patients discontinued distance correction (spectacle or rigid contact lens) and 4 patients changed from rigid contact lenses to glasses. In the moderate KC group, the mean BPE was −0.3 D but again the BPE range was wide (from 3.2 D to −3.8 D). All patients in the moderate KC group required distance glasses or contact lens correction after the surgery. Seventeen eyes were enrolled in the severe KC group where optical biometry actual Ks values were used for 8 eyes and standard K values for 9 eyes. Where actual K values were used, the authors found a BPE mean of 6.8D (range 0.2–17D). Conversely, refractive outcome was more predictable for the 9 patients where standard K values (43.25D) were used resulting in a mean BPE of 0.6 D (range: 6.2D to −5.8D). In all cases the SRK-T formula was used with a refractive target of mild myopia. This study offers a helpful description of possible refractive outcomes after cataract surgery according to KC grade aiding ophthalmologists with pre-operative planning and patient counselling. Leccisotti [20] published data on 34 consecutive eyes affected by KC grade I or II that underwent refractive lens exchange. Holladay 2 formula was used for all cases and K measurements were taken from topography over the central 3mm of the cornea. Preoperative mean spherical equivalent (SE) was -11.0 ± 4.65 D (range: -5.75 to -22 D). The author performed intraoperative autorefraction followed by immediate IOL exchange in cases with spherical refractive error 1.5 D (7 eyes, 26%). IOL exchange was performed in 25% of eyes with stage I disease compared with 50% of eyes with stage II disease, suggesting reduced accuracy of biometry in the latter group.

## 3 TORIC INTRAOCULAR LENS

Toric IOLs have been designed to correct astigmatism after cataract surgery. Literature regarding the use of toric IOLs in keratoconic eyes remains scarce with few published papers demonstrating the use of toric IOLs in KC patients with good outcomes.

Hashemi *et al.* [21] investigated the changes in refraction, uncorrected and corrected visual acuity of 23 eyes of 17 patients affected by stable KC who received a toric IOL implant during standard cataract surgery. The severity of KC was classified as mild (10 eyes), moderate (10 eyes) and severe (3 eyes) disease when the Kmax was <48 D, between 48-52 D and >52 D respectively. The Hoffer Q, SRK II, Holladay I, and SRK/T formulas were used for patients with AL 22mm, 22-24.5 mm, 24.5-26 mm, and 26 mm, respectively using the IOL Master (Carl Zeiss Meditec, Germany) biometry.

 Authors found that uncorrected and best-corrected visual acuity (BCVA) improved significantly after surgery in the mild and moderate group. In addition, there was a mean BCVA improvement of 2.25 ± 1.50 lines postoperatively in the 3 eyes of severe KC compared with preoperative BCVA. In a similar study, Nanavaty *et al.* [22] reported 83% of their patients achieving BCVA of 20/40 or better in their series of 9 patients (12 eyes) with mild-moderate stable KC after cataract surgery. In addition, mean uncorrected distance visual acuity improved significantly from 20/400 preoperatively to 20/40 postoperatively. Authors highlighted that the most important predictive factor for a successful outcome with toric IOL implant in keratoconic eyes is good preoperative visual acuity with spectacle correction. A history of contact lens dependency, surface irregularity, and corneal scarring over the visual axis are predictive factors for poor visual outcome.

## 3.1 Intraoperative Considerations

Modern phacoemulsification with IOL implantation in keratoconic eyes can be technically challenging with the difficulty of surgery being related to the grade of KC disease. Early KC can be managed in a similar manner to normal cataract surgery; however, severe KC with steep meridians and possible corneal scars can be troublesome. In modern cataract surgery the astigmatism induced by the single main clear corneal incision has become negligible. However, when the main incision is performed in a cornea with abnormal structural properties as in keratoconic eyes, post-operative K values and corneal shape can change in an unpredictable manner. Therefore, cataract main incision sites should be planned during preoperative examination according to the peripheral corneal thickness rather than the astigmatism axis. In the case of infero-temporal cone, the main incision should be placed superiorly or supero-temporal. Vice versa, in rare cases of rare superior steep cone the main incision should be placed temporally.

Planning the location of the incision should also consider the presence of corneal scarring which is common in advanced KC. In these cases, the main incision should be placed 90 degrees apart from the scar location. In cases of inferior scarring, a superior approach is not advised as this will lead the phaco-probe to point inferiorly (underneath the scar) rendering all manoeuvres more challenging due to the reduced intraocular visibility. Additionally, keratoconic corneas are thinner and floppier and therefore clear corneal wounds are more prone to leak after surgery. Thus, a well-constructed 2 steps sclero-corneal is advisable to reduce the risk of post-operative wound leak and to induce less change in corneal shape.

 The use of a corneal suture to secure the wound can be advisable. In the presence of diffuse scars, the surgeon should expect a worse view under the surgical microscope compared with the slit lamp examination. This is due to the different light sources employed: a sharp oblique light at the ophthalmic slit lamp versus a central diffuse light under the surgical microscope. The latter will cause a light scattering together with shadows and reflections that will make the surgical steps more difficult. To improve intraocular visibility and reduce image distortion due to the corneal irregularity it can be useful to spread a dispersive ophthalmic viscoelastic device such as the hydroxypropyl methylcellulose (HPMC) gel onto the cornea. This will improve the epithelial hydration rendering the corneal surface more regular and provide a mild magnification of the intraocular view [23]. After making the corneal incisions, even in cases of good red reflex the use of capsular staining dye is recommended to enhance capsular visualisation during continuous curvilinear capsulorhexis.

 This will make the capsulorhexis and subsequent surgical manoeuvres easier by increasing the contrast between intraocular structures and the perception of depth otherwise reduced by the corneal refractive multifocality. In order to overcome the poor visibility during cataract surgery in keratoconic eyes, Oie *et al.* [24] described a new technique using a rigid gas permeable contact lens during the procedure. After sterilizing the contact lens with low temperature (55**°**C for 210 minutes) and ethylene oxide gas, the authors applied the lens onto the corneal surface applying a cohesive viscosurgical device to fill the eye-contact lens interface. Although only 2 cases were described, the authors found a significant improvement in visibility at each stage of cataract surgery.

During the surgery, it is also important to attempt to minimize the intraocular pressure by modifying the phacoemulsification machine parameters to reduce stress on the cornea.

## CONCLUSION

Cataract surgery in keratoconic eyes is technically more challenging compared with normal eyes. When elderly patients develop visual deterioration, a thorough assessment should be performed to determine the cause. The KC progression tends to stop when patients enter their 30’s and consequently, its progression is extremely unlikely after 50. Thus, a careful evaluation of corneal topography measurements within suitable repeatability limits is required [25].

Keratoconic eyes tend to be a very unstable optical system compared with normal eyes and can be much more sensitive to lenticular changes [26]. We suggest that in keratoconic eyes small changes in lens clarity can significantly impact upon the visual axis which could be largely responsible for visual symptoms rather than an actual progression in the KC. This is important because as mentioned previously keratoconic patients tend to develop lens opacification earlier on in life [10].

Therefore, when ophthalmologists decide to proceed with cataract surgery, accurate IOL selection presents a significant challenge. Existing methods to determine the IOL power are not entirely suitable for keratoconic eyes. Furthermore, results vary for different grades of the disease. In mild keratoconus, better results can be obtained compared with severe KC and toric IOLs can be carfully considered.

As showed by Watson *et al.* [19], the use of actual biometry derived K values aiming for a low myopia refractive target can give acceptable results for mild to moderate KC. However, in eyes with mean K >55D the use of standard K values and low myopia refractive target should be considered. Consideration should also be taken regarding the options for postoperative refraction correction.

 If a CL had been worn prior to the development of the cataract, continued use is necessary after surgery to maximise visual function. Without continued CL wear, the best possible visual acuity post surgery can only be expected to be equal to the best spectacle corrected acuity prior to the development of the cataract. A large majority of keratoconic patients, with more than just mild disease are dependent on CL for useful vision, which would remain the case post surgery since the IOL can only correct the spherical refractive error.

 A low myopic post-operative refractive target is preferable compared with a hyperopic refractive outcome not only to enable unaided near vision but also to facilitate scleral CL wear. This is related to the optics of a scleral lens. The pre-corneal tear fluid reservoir usually works as a very high power minus lens, sometimes up to –15.00 D. Thus, in the case of a postoperative hyperopic refractive outcome, the use of scleral CLs would be more difficult. This is because positive powered scleral CLs are less satisfactory and they require greater central thicknesses with smaller front optic zone diameter resulting in a CL that is usually more mobile in situ and with higher degrees of optical aberration.

In conclusion, the grade of KC is crucial in determining the strategy of cataract surgery. Due to the scarcity of literature for IOL selection and the difficulty in obtaining reliable keratometry, the most important step in planning the cataract surgery in the keratoconic eye is not only the IOL selection but also patient counselling especially the discussion of the postoperative outcomes. In light of generally unpredictable post-operative refractive results, patients using spectacles preoperatively may require contact lenses after surgery and vice versa. Where there is an unacceptable refractive surprise IOL exchange or piggyback lens placement may be necessary. With appropriate patient selection, toric IOLs are justified to be used in cases with stable non progressive keratoconus and where there is a good level of spectacle corrected visual acuity.
